# Thermal, Mechanical, and Electrical Stability of Cu Films in an Integration Process with Photosensitive Polyimide (PSPI) Films

**DOI:** 10.3390/nano13192642

**Published:** 2023-09-26

**Authors:** Ruhan E. Ustad, Vijay D. Chavan, Honggyun Kim, Min-ho Shin, Sung-Kyu Kim, Kyeong-Keun Choi, Deok-kee Kim

**Affiliations:** 1 Semiconductor Systems Engineering, Sejong University, Seoul 05006, Republic of Korea; 2 Electrical Engineering and Convergence Engineering for Intelligent Drone, Sejong University, Seoul 05006, Republic of Korea; 3 National Institute for Nanomaterials Technology (NINT), Pohang University of Science and Technology (POSTECH), Pohang 37673, Republic of Korea

**Keywords:** polyimide, photosensitive, Cu RDL, resistivity, advanced packaging

## Abstract

Photosensitive polyimides (PSPIs) have been widely developed in microelectronics, which is due to their excellent thermal properties and reasonable dielectric properties and can be directly patterned to simplify the processing steps. In this study, 3 μm~7 μm thick PSPI films were deposited on different substrates, including Si, 50 nm SiN, 50 nm SiO_2_, 100 nm Cu, and 100 nm Al, for the optimization of the process of integration with Cu films. In situ temperature-dependent resistance measurements were conducted by using a four-point probe system to study the changes in resistance of the 70 nm thick Cu films on different dielectrics with thick diffusion films of 30 nm Mn, Co, and W films in a N_2_ ambient. The lowest possible change in thickness due to annealing at the higher temperature ranges of 325 °C to 375 °C is displayed, which suggests the high stability of PSPI. The PSPI films show good adhesion with each Cu diffusion barrier up to 350 °C, and we believe that this will be helpful for new packaging applications, such as a 3D IC with a Cu interconnect.

## 1. Introduction

Semiconductors and power-efficient devices are receiving more attention, which is due to their extremely efficient operational properties and extraordinary working capabilities, with worldwide advancement [1,2,3]. The semiconductor industry is in need of materials with low cost, negligible RC delay, and reduced cross-talk for next-generation integration systems [4,5,6]. Thus, industries are breaking records due to advanced technology, mobility, and connectivity, along with the digital transport of smart devices with outstanding performance and low power consumption [7,8]. A lot of efforts have succeeded in integrating the number of transistors per unit area with operational frequency over the decades. Future silicon (Si)-based technology will therefore find challenges concerning meeting the further expected requirements. However, Moore’s law settled its limitations beyond the technology node, so companies have been lobbying for novel breakthroughs in electronic devices [9,10]. Higher-level assembly, such as a system in packaging-SiP, which will improve the device characteristics and enhance functionality, is globally recommended. 

Heterogeneous integration (HI) is very well known as the aggregation or integration of various components in higher-level assembly. Hence, HI can deal with the above-mentioned threat with the advanced packaging of all the components on one chip. Copper (Cu) plays an excellent and crucial role in HI and advanced packaging due to its high conductivity, integration density, and direct connectivity with the advanced packaging of all the components on one chip [11,12,13]. However, the dimension scaling of the device may cause several influences on the Cu redistribution line. Cu electromigration (EM) and diffusion into the surrounding components are critical issues for reliable next-generation HI technology [14,15,16]. The investigation is focused on the Cu diffusion barrier and low-k materials that are capable of blocking the Cu EM/diffusion and protecting the device to avoid uncertainty in performance [17,18,19,20,21,22]. Several low-k materials, which include silicon dioxide (SiO_2_), polybenzoxazole (PBO), benzocyclobutane (BCB), and photosensitive polyimide (PSPI), have been used [23,24,25]. 

PSPI is favorably adopted among all the low-k materials because of its advantage of photolithographic properties for making simplified patterns with high precision and accuracy [26]. However, its required quality as a final product to maintain its structural integrity makes it suitable for applications where long-term performance is essential [27]. The quality of a low-k material can be assessed through its thermal, mechanical, and electrical properties. Various studies have been reported which tested the mechanical properties of PSPI [28,29,30]. However, very few have used the nanoindentation technique to do so. As this is a modern and precise technique, there is still a gap in the literature on PSPI. The thermal stability of PSPI has been studied by making its composites with boron nitride [31] and carbon-based materials [32,33]. These studies report the thermal stability of only PSPI. However, knowledge of thermal stability for advanced packaging is lacking. Considering low-k materials, PSPI has very low electrical conductivity and has previously been studied [34]. However, the study of the diffusion of Cu migration in the low-k materials has not been depicted in the literature. 

Considering the above, in this study, 3 μm~7 μm thick PSPI films were deposited on Si, 50 nm SiN, 50 nm SiO_2_, 100 nm Cu, and 100 nm Al substrates for the optimization of the process of integration with Cu films. To understand the feasibility of advanced packaging, the above different substrates were chosen. The changes in the resistivity of the 70 nm thick Cu films on different dielectrics with 30 nm thick diffusion films, such as Ta, Co, and W films, in a N_2_ ambient were measured by using a four-point probe in in situ temperature-dependent resistance measurements. The change in thickness after annealing was studied at a higher temperature range from 325 °C to 375 °C. The PSPI films show good adhesion with each Cu diffusion barrier up to 350 °C, and we believe that this will be helpful for new packaging applications, such as a 3D IC with a Cu interconnect. 

## 2. Materials and Methods

### 2.1. Materials and Characterization

The PSPI material was bought from Toray (PW-1500 series). The deposition of PSPI was carried out using a spin coater. The annealing process was performed using a tube furnace. For the thickness measurements, a KLA Tencor D-600 stylus profilometer was used. The microscope images were recorded using an OLYMPUS U-MSSP4 microscope. The electrical measurements were conducted on an Agilent 3600 probe station. The resistivity was measured by fabricating a Cu/PSPI/SiO_2_/Si structure. The mechanical property testing was carried out on a nanoindentation tester (NHT3 Anton Paar).

### 2.2. Substrate Preparation

Before photopatterning of PSPI, different substrates like Cu, SiN, and SiO_2_ were prepared. Onto Si films, 50 nm of SiN and 50 nm of SiO_2_ were deposited using the PECVD method. For better adhesion, hexamethyldisilazane (HMDS) was spin-coated on the 150 mm and/or 200 nm diameter wafers at 110 °C for 60 s. by using a commercially available track (TEL, Mark7). These substrates were further used for the spin coating and photopatterning of PSPI.

### 2.3. Spin Coating and Photopatterning of PSPI

**Figure 1:**
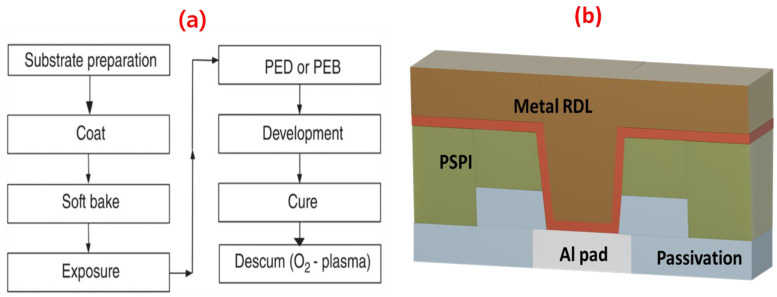
(**a**) Process flow chart for the PSPI integration and (**b**) a simplified cross-sectional schematic diagram of the redistribution layer (RDL).

An aligner and a stepper (i-line, ASML, PAS5500 200B) were used for all of the PSPI patterning processes after a soft bake at 120 °C for 6 min. The final targeted film thickness was split in the range from 3 μm to 7 μm, and the 7 μm thick prebaked film for patterning was exposed at approximately 550 mJ/cm^2^. Figure 1a shows the process of PSPI film spin coating and photopatterning. An RDL layer, which is a redistribution layer, consists of a metal layer and a PSPI dielectric layer, which is shown in Figure 1b. The PSPI films were deposited using the PSPI integration process, which is shown in Figure 1b. Table 1 shows the photopatterning of PSPI onto different substrates at different thicknesses.

## 3. Results and Discussion

**Figure 2:**
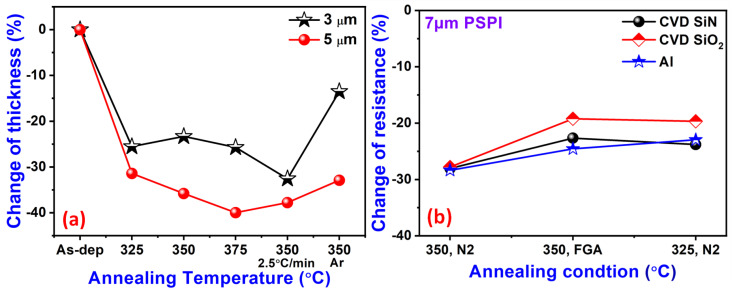
(**a**) Thickness of the PSPIs after the annealing on 100 nm Cu films and (**b**) the change in resistance (%) of the 7 μm target-thickness films at each annealing condition on 50 nm SiN, CVD SiO_2_, and 100 nm Al substrates.

The annealing of PSPI films under different conditions can affect different properties of the polymer material. Figure 2a illustrates the change in thickness as a function of the annealing temperature for PSPI deposited on the 100 nm Cu films. Other annealed PSPI (3 and 5 µm) samples showed reductions in the thickness compared to the as-deposited sample. A change in the dimensions due to the shrinkage of the PSPI can be observed with different annealing conditions [35,36]. However, the annealing at 350 °C with a temperature ramp rate of 2.5 °C/min under a N_2_ ambient displays a similar behavior for both the 3 and 5 µm PSPI samples. This may be attributed to the slow heating rate, which stabilizes the damage that occurs due to fast heating. The changes in resistance of the 7 μm target-thickness PSPI films under different annealing conditions on 50 nm SiN, CVD SiO_2_, and 100 nm Al substrates were studied. The resistance changes of the PSPI sample under FGA (350 °C) and a N_2_ ambient for 325 °C and 350 °C (2.5 °C/min) were measured. The N_2_ ambient shows negligible change in resistance at 350 °C. However, the FGA environment causes an increase in the resistance of the SiO_2_ substrate. The resistance change of the Al substrate is not constant at all. The differences in the thermal and electrical conductivity of the base substrates may have caused the observed change in the resistance of the Cu films. 

**Figure 3:**
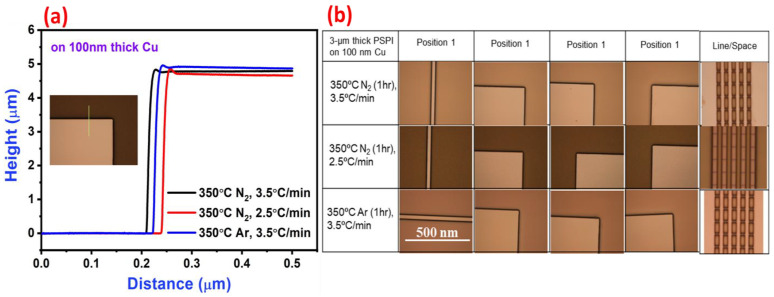
(**a**) Measured PSPI thicknesses after annealing of 5 μm thick film on 100 nm thick Cu at three different temperatures (image inside shows the path followed by stylus profiler tip during thickness measurement) and (**b**) optical images of the pattern defined on long length line (position 1), 100 μm width pads (positions 2–4), and line (10 μm)/space patterns.

**Figure 4:**
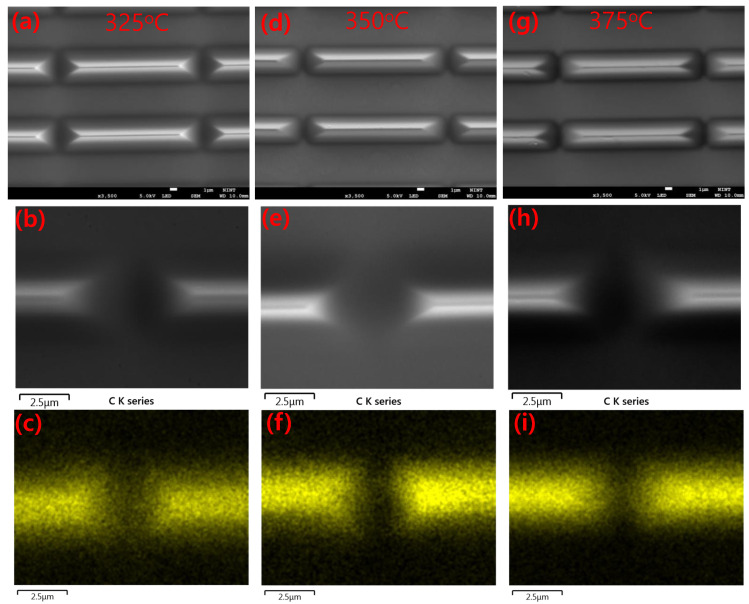
Low- and high-resolution SEM micrographs and EDX spectra of Cu 3 μm line/space in N_2_ annealing at 325 °C (**a**–**c**), 350 °C (**d**–**f**), and 375 °C (**g**–**i**).

The annealing of PSPI films in different temperatures strongly affects the film thickness. For thickness measurements, stylus profilometer measurements were conducted on 5 μm thick PSPI, as shown in Figure 3a. The shrinkage in the thickness can be seen very clearly from the above data. Less variation in the thickness was observed even at a low scan rate in our experiments. Figure 3b shows the microscope images of annealed films, which were conducted to see the effect of heating on the films. Our purpose was to observe the damage that was created in the film due to annealing. The observed optical image demonstrates the distance between two pattern lines at position 1, in which no damage occurred. The microscope images of 100 μm pads at three different positions show no damage due to any type of heating of the films. We patterned the Cu films with a 10 μm line/space of PSPI, and the microscope image of this line/space pattern shows the stability of PSPI on Cu film after high-temperature annealing. For thorough analysis, we recorded SEM images with EDX mapping of Cu with a 3 μm line/space (Figure 4). As can be seen through the EDX mapping of carbon elements, even after the use of different annealing temperatures in the N_2_ environment, carbon from PSPI is not spread through Cu films. This proves the high stability of PSPI. Further, the stability in thicknesses, which is due to different annealing conditions, suggests that PSPI can be used in fan-out wafer-level packaging.

**Figure 5:**
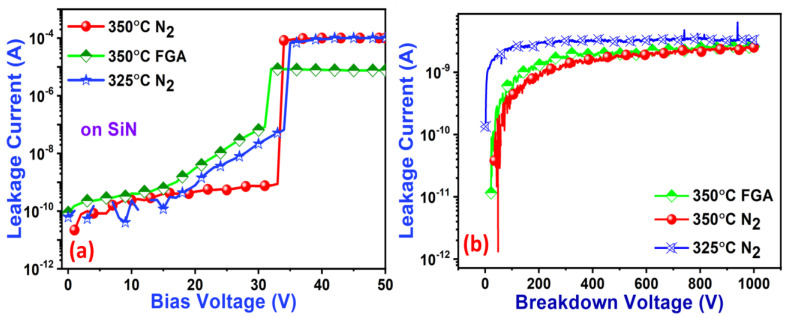
(**a**) Leakage current as a function of voltage with different annealing conditions on 50 nm SiN and (**b**) 7 μm PSPI/50 nm SiN substrates.

Leakage current flowing through the device can reduce the performance of any electrical device. The reduction in the leakage current is very important for advanced packaging [37]. Researchers and manufacturers are continuously working on developing new materials and optimizing the manufacturing processes to mitigate the disadvantages of the leakage current in advanced packaging [38]. The leakage currents of the 50 nm SiN (a) and PSPI (5 μm)/SiN(50 nm) films are displayed in Figure 5. The SiN films show different leakage behavior with different annealing conditions. However, the SiN sample annealed at 350 °C (2.5 °C/min) gives a leakage current of 8.8 × 10^−10^ A/cm^2^, which is lower than that for the other annealed conditions. Figure 5b demonstrates the breakdown voltage of the 7 μm PSPI/50 nm SiN sample. The breakdown voltage of 0 to 1000V was applied to the prepared samples under FGA (350 °C) and a N2 ambient for 325 °C and 350 °C (2.5 °C/min) annealing conditions.

**Figure 6:**
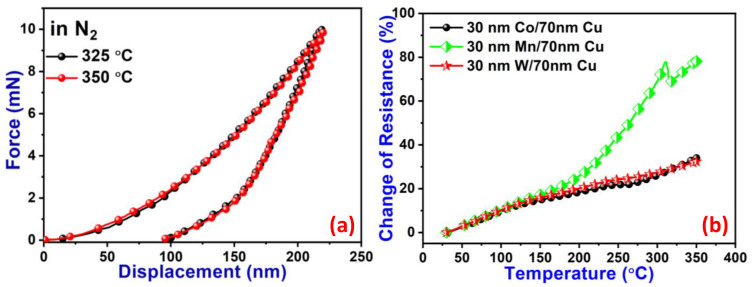
(**a**) Indentation load as a function of displacement for the different annealing temperatures on Cu substrates and (**b**) the change in resistance as a function of measuring temperature.

It is essential to have a low-k dielectric with good-quality mechanical properties, which keeps the microchips stable during pressurized bonding. We implemented the nanoindentation technique to study the mechanical properties of patterned PSPI films, which are shown in Figure 6a. Annealing at a higher temperature does not change the mechanical properties of the PSPI films, which shows the stability of PSPI at a higher temperature. The Young’s modulus of the film annealed at 325 °C is 9.485 GPa with a hardness of 0.468 GPa, and that of the film annealed at 350 °C is 9.529 GPa with a hardness of 0.479 GPa. The area between the loading and unloading curve represents the energy lost during plastic deformation [39,40]. The average energy lost during plastic deformation was 1.891 × 10^−3^ mJ, which shows that the elasticity of the films was considerably lower. The resistance of a metal increases with increasing temperature, and the resistance of Cu wildly increases. However, forming interfaces with different metals can reduce this. On the other hand, metal interfaces are formed in advanced packaging systems in which metal pads are used below Cu. To test the electrical stability of Cu at different temperatures over different metal interfaces, we fabricated interfaces of Cu with Co, Mn, and W for a more detailed study. Figure 6b shows the increase in resistance with increasing temperatures. The resistance of Co/Cu and W/Cu smoothly increased; however, the resistance of Mn/Cu increased drastically after 175 °C. This sudden increase in resistance may be due to the presence of defects inside the films [41]. These interfaces can be used for the diffusion barrier for Cu by considering the Cu interconnects [42].

## 4. Conclusions

We studied the thermal, electrical, and mechanical stability of PSPI for advanced packaging systems. The extremely low shrinkage in the film thickness after the use of different annealing temperatures suggests that PSPI is stable at higher temperatures (350 °C). The resistance changes of Cu on different substrates under different ambient annealing conditions were studied. The stability of the resistance change in FGA and N2 ambients shows the industrial applicability of PSPI. The mechanical properties, which were tested using nanoindentation, showed that PSPI possessed a high Young’s modulus of 9.52 GPa. There was no leakage current found until 30 V. Considering these results, it can be proved that the use of PSPI in advanced packaging systems is feasible, and it can also be used for reducing Cu electromigration.

## Figures and Tables

**Table 1:** The photopatterning of PSPI onto different substrates at different thicknesses.

Substrate	Thickness of PSPI	Cu Thickness	Curing
Cu (100 nm)	3 and 5 μm	300 nm	As kept
Cu (100 nm)	3 and 5 μm		325 N_2_ (3.5 °C/min)
Cu (100 nm)	3 and 5 μm		350 N_2_ (3.5 °C/min)
Cu (100 nm)	3 and 5 μm		375 N_2_ (3.5 °C/min)
Cu (100 nm)	3 and 5 μm		350 N_2_ (2.5 °C/min) (slow)
Cu (100 nm)	3 and 5 μm		350 Ar (3.5 °C/min)
SiN (50 nm)	7 μm		325 and 350 N_2_ and 350 FGA (3.5 °C/min)
SiO_2_ (50 nm)	7 μm		325 and 350 N_2_ and 350 FGA (3.5 °C/min)
Al (100 nm)	7 μm		325 and 350 N_2_ and 350 FGA (3.5 °C/min)

## Data Availability

No new data were created.
